# Association Analysis on Recurrence of Bacterial Vaginosis Revealed Microbes and Clinical Variables Important for Treatment Outcome

**DOI:** 10.3389/fcimb.2019.00189

**Published:** 2019-06-11

**Authors:** Bingbing Xiao, Chunyan Wu, Wenfeng Song, Xiaoxi Niu, Nan Qin, Zhaohui Liu, Qian Xu

**Affiliations:** ^1^Department of Obstetrics and Gynecology, Peking University First Hospital, Beijing, China; ^2^Realbio Genomics Institute, Shanghai, China; ^3^Shenzhen Jinrui Biotechnology, Co., Ltd., Shenzhen, China

**Keywords:** bacterial vaginosis (BV), recurrence, 16S rRNA gene, metronidazole, *Lactobacillus*, *Enterococcus*

## Abstract

To investigate the parameters associated with post-treatment recurrence of bacterial vaginosis (BV), clinical factors and vaginal microbiota were examined and analyzed for BV patients who received standard metronidazole therapy. The variables associated with BV recurrence included clinical factors of past BV history, use of intravaginal device, and D7 Nugent score as well as many microbial genera, with *Lactobacillus, Enterococcus, Ureaplasma*, and *Aerococcus* being the top contributors. Co-occurrence network analysis showed that whereas overwhelming majority of interbacterial interactions were positive, negative interactions were present and connected mostly to *Lactobacillus, Enterococcus*, and to a less extent *Ureaplasma*, suggesting the importance of interbacterial antagonism for treatment outcome. The patients who were cured and recurrent also exhibited clear differences in the species composition of *Lactobacillus*: although *L. iners* remained the dominant species at all time points, *L. crispatus, L. gasseri*, and *L. jensenii* displayed apparent differences in relative abundance between the cure and recurrent groups. Based on these results, we developed a 5-component panel comprising *Enterococcus, L. crispatus, Ureaplasma, Aerococcus*, and *L. jensenii* for predicting recurrence using D7 data and showed that it generated the specificity, sensitivity, and AUC values of 0.80, 0.66, and 0.73 for the discovery cohort and 0.80, 0.67, and 0.69 for the validation cohort. Our findings highlighted key microbial components for BV recurrence and suggested that they could be used to monitor the treatment outcome.

## Introduction

Bacterial vaginosis (BV), a condition where vaginal lactobacilli are subject to great levels of replacement by predominantly anaerobic microorganisms, is a common infection in the lower female genital tract that affects 10–30% of women in many countries (Koumans et al., 2007; Ravel et al., 2011; Ma et al., 2012; Xiao et al., 2016). Women with BV are more likely to experience gynecological and obstetric complications, including amniotic fluid infection, chorioamnionitis, postpartum endometritis, preterm delivery, as well as increased susceptibility to HIV and other sexually transmitted infections (Eschenbach, 2007; McClelland et al., 2008; Oliver and Lamont, 2013). Diagnosis of BV remains a challenge in primary medical settings and involves identification of multiple clinical signs, including a gray homogeneous vaginal discharge, positive “whiff test” (fishy odor after potassium hydrogen treatment), vaginal pH >4.5, and presence of clue cells (i.e., vaginal epithelial cells with bacteria adherent to the surface) (Marrazzo et al., 2010). In addition to the diagnostic difficulties, BV is also a hard-to-treat disease. Currently, first-line therapy for BV uses metronidazole and clindamycin (Ferris et al., 1995), both of which have considerable rates of recurrence (57–90% at 1 to 3 months and 34–51% at 1 year) (Fischbach et al., 1993; Livengood et al., 1999; Bradshaw et al., 2006; Koumans et al., 2007; Bunge et al., 2009; Xiao et al., 2016).

Given the recalcitrant and dysbiotic nature of BV, we argue that it is clinically important to identify recurrence-associated biomarkers or traits, which may help to timely monitor treatment progress and facilitate personalized therapy to improve the cure rate. Some clinical and demographic variables have been reported to correlate with post-treatment recurrence of BV, including a past history of BV and a regular sex partner (Bradshaw et al., 2006). In addition, it was reported that several *Lactobacillus* species, including *L. gasseri, L. brevis*, and *L. acidophilus*, could reduce BV recurrence (Neri et al., 1993; Shalev et al., 1996; Anukam et al., 2006), although such findings were controversial (Fredricsson et al., 1989; Hallen et al., 1992; Eriksson et al., 2005). Of note, these studies all used simple, traditional methods of clinical microbiology, which are inadequate to characterize the complex and dynamic composition of vaginal microbiota (Ravel et al., 2011).

In this study, we recorded a set of clinical factors and examined vaginal microbiota in two clinical groups of Chinese BV patients (i.e., cure vs. recurrence) over an 1-month course before and after a standard intravaginal metronidazole therapy, whereby recurrence-associated parameters were investigated. Our data indicated that post-treatment recurrence of BV is associated with several clinical and microbial variables. We propose that personalized treatment based on vaginal microbial composition and/or clinical information may help improve the therapeutic outcome of the disease.

## Materials and Methods

### Diagnosis, Treatment, and Visits

Bacterial vaginosis was diagnosed if the Nugent score was between 7 and 10 in patients with relevant clinical symptoms (Marrazzo et al., 2010). Using the Nugent method (Nugent et al., 1991), all vaginal smears were scored independently by two experienced microscopists who were blind to the patients' clinical information. Vulvovaginal candidiasis was diagnosed if the vaginal fluid was found to exhibit yeast forms in patients having the typical symptoms. Women with bacterial vaginosis were treated with a 5-day regimen of intravaginal metronidazole gel (37.5 mg daily) and were asked to return 1 month later for a test-of-cure examination (Marrazzo et al., 2008; Fredricks et al., 2009). Cure of bacterial vaginosis was defined by lack of a significant number of clue cells, normalization of pH (4.5 or less), and a Nugent score of 0–3 (normal flora) (Marrazzo et al., 2010). The participants were surveyed for BV-related symptoms, such as abnormal vaginal discharge and odor. Treatment failure or BV recurrence was defined by a Nugent score ≥7. Women who were found to have bacterial vaginosis at the test-of-cure visit were treated with another course of intravaginal metronidazole of the same dosage.

### Study Population

This study was approved by the Ethics Committee of Peking University First Hospital, Beijing, China. The study design is illustrated in Supplementary Figure 1. Briefly, women were diagnosed with BV and treated in the Department of Obstetrics and Gynecology, Peking University First Hospital. Written informed consent was obtained from all subjects. Upon diagnosis, the patients were treated with a single 5-day regimen of intravaginal metronidazole gel (37.5 mg daily) and were asked to return 6–8 days later and 1 month later for examination of the outcome.

The discovery cohort was recruited between September 2015 and July 2016, during which a total of 351 women with BV were screened for their eligibility of this study. The exclusion criteria included age <18 years or above 55 years old, pregnancy, menstruation, sexual intercourse within 24 h, use of antibiotics in the last month, use of intravaginal products in the last 24 h, and presence of yeast on Gram stain or *Trichomonas vaginalis* infection. Of the 109 qualified subjects, 67 (61.47%) returned for the examination visit after 1 week (8.3 ± 0.9 days) and the test-of-cure visit after 1 month (31.2 ± 7.0 days) (Supplementary Table 1). Based on their Nugent scores (Supplementary Table 1) of the test-of-cure assessment (Marrazzo et al., 2008; Fredricks et al., 2009), 47 women (70.1%) were determined to be cured, whereas 20 (29.9%) were determined to be recurrent. These 67 subjects constituted the discovery cohort to investigate recurrence-associated variables and generate a prediction model of recurrence. To validate the prediction model, 30 qualified females were recruited between December 2018 and January 2019.

### Collection of Vaginal Samples and DNA Extraction

Vaginal samples were collected at the first visit (D0), 7 days later (D7), and 1 month later (D30). At each visit, two vaginal swabs were placed into the vagina at a standard anatomical site (lateral vaginal wall) (Marrazzo et al., 2010). The first vaginal swab was processed for gram staining and the other used for genomic DNA extraction.

Vaginal specimens were coded, stored and processed for bacterial genomic DNA extraction using the method described by Ling et al. (2010). Genomic DNA was extracted using the QIAamp DNA Mini Kit (QIAGEN, Germany). Briefly, 20 μl proteinase K solution (20 mg/ml) and 100 mg zirconium beads (0.1 mm) were added to the pellet. The mixture was agitated three times on a Mini-Beadbeater (FastPrep, Thermo Electron Corporation, MA, USA); buffer AL was added to the mixture, which was then incubated for 10 min at 70°C. Next, 200 μl ethanol (96%) was added, before this mixture was loaded onto the QIAamp Mini spin column and centrifuged at 8,000 *g* for 1 min. The column material was washed with the first washing buffer (buffer AW1, 500 μl) and the second washing buffer (buffer AW2, 500 μL). Finally, DNA was eluted with 100 μL buffer AE. The integrity and size of the extracted DNA were confirmed by electrophoresis on 1% agarose gel containing 0.5 mg/ml ethidium bromide. The DNA concentration was determined using a NanoDrop ND-2000 spectrophotometer (Thermo Electron Corporation, MA, USA).

### 16S rRNA Gene Sequencing and Data Processing

For analysis of samples in the discovery cohort, the 16S rRNA gene hypervariable V1-V3 region was amplified using the primers 27F (5′-AGAGTTTGATCCTGGCTCAG-3′) and 533R (5′-TTACCGCGGCTGCTGGCAC-3′). Amplicon pyrosequencing was performed using standard 454/RocheGS-FLX protocols (Margulies et al., 2005). For analysis of samples in the validation cohort (D7 samples only), the 16S rRNA gene hypervariable V3-V4 region was amplified using the primers 341F (5′-CCTACGGGRSGCAGCAG-3′) and 806R (5′-GGACTACVVGGGTATCTAATC-3′). After preparation of library, these tags were sequenced on MiSeq platform (Illumina, Inc., CA, USA) for paired-end reads of 250 bp. After the sequencing, all reads were sorted, screened, and filtered to ensure quality and length as previously described (Hamady et al., 2008). The denoised reads were dereplicated to a unique sequence and were then sorted by abundance and subjected to OTU clustering at 97% similarity. To search the closest species, the representative sequence of each OTU was classified using the RDP classifier and stored at the Ribosomal Database Project (Cole et al., 2009). Identification of *Lactobacillus* species was performed as described previously (Ravel et al., 2011).

### Statistical Analysis

Rarefaction curves were created to ensure adequate sequencing depth for each sample. To assess the significance of differences between groups, we performed the Kruskal test in R-3.2.3 (http://cran.r-project.org) for the relative abundance of each bacterial group as determined using sequencing of the 16S rRNA gene. Alpha diversity was measured by the observed species diversity index and Shannon diversity index. Unweighted UniFrac and principal coordinates analysis (PCoA) were used to analyze differences in beta diversity. Wilcoxon rank sum test was performed to identify the phylotypes differentially abundant in the cure group and recurrence group. For analysis of clinical, demographic and behavioral factors, rank sum test was used for continuous variables; Chi-square test was used for categorical variables; Fisher exact test was used for discrete variables. Random forest analysis and Extreme Gradient Boosting (XGBoost) algorithm (Chen and Guestrin, 2016) were employed to determine the importance ranking of microbial taxa in BV recurrence.

### Analysis of Co-occurrence Network

To construct the meta-community co-occurrence networks, we first removed genera with relative abundances positive in <10 samples. The Spearman correlation coefficients between genera were computed using R; all the *P*-values were adjusted for multiple testing using the Benjamini and Hochberg false discovery rate (FDR) controlling procedure. Based on FDR (<0.05) adjusted *P*-values for correlation, we constructed the co-occurrence networks for the two treatment groups. The co-occurrence networks were visualized by Gephi.

### Accession Number

The sequence data in this study have been deposited in NCBI under BioProject number PRJNA433569.

## Results

### General Information and Clinical Characteristics of the Study Subjects

Most of the analyses in this study were performed on the subjects of the discovery cohort except for the validation of a prediction model, which involved the validation cohort. The subjects of the discovery cohort had an age range of 18–53 years old and a mean age of 35.8 (± 8.2) years old; their demographic and behavioral traits are summarized in Supplementary Table 1. The subjects of the validation cohort had an age range of 22–55 years old and a mean age of 37.1 (± 8.7) years old (Supplementary Table 2); there was no age difference between the two cohorts (*P* > 0.05). In the discovery cohort, 26 (55.3%) women reported a past history of BV; 5 women (10.6%) underwent a previous uterine cavity surgery; 20 women (42.6%) had used intrauterine device. All individuals were shown to exhibit a high Nugent score (7–10) in the first visit and had other concurrent symptoms (e.g., pH >4.5, aberrant odor, and discharge) associated with bacterial vaginosis.

All participants completed a single 5-day regimen of intravaginal metronidazole gel (37.5 mg daily), a follow-up examination 6 to 8 days later, and a test-of-cure examination 1 month later. They were sampled for their vaginal microbiota at the three time points: day 0 (D0, start of the regimen), day 7 (D7, first follow-up visit), and day 30 (D30, second follow-up visit for test-of-cure examination). Based on the Nugent scores on D30, the 97 patients were divided into two clinical groups, namely the cure group (47 individuals in the discovery cohort and 15 in the validation cohort) and the recurrence group (20 individuals in the discovery cohort and 15 in the validation cohort), respectively.

### Dynamics of Vaginal Microbiota in Subjects of the Discovery Cohort

**Figure 1:**
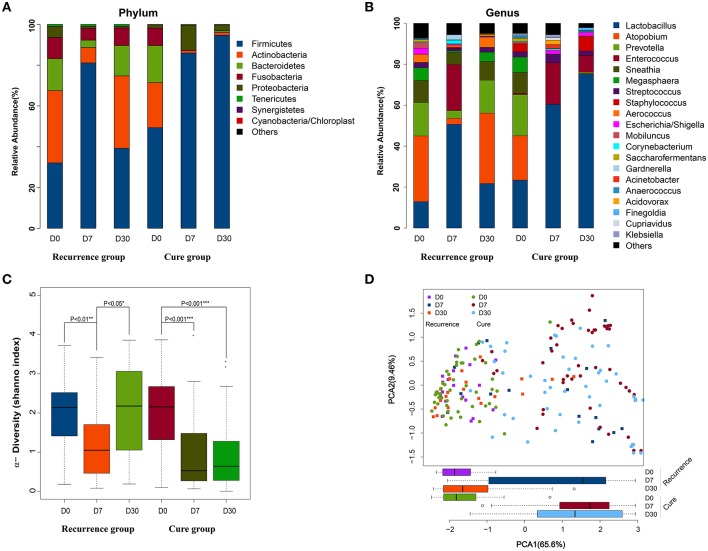
Comparison of vaginal microbiota at different time points between the cure group and recurrence group. **(A)** Top 20 abundant phyla; **(B)** top 20 abundant genera; **(C)** α-diversity (Shannon index); **(D)** PCoA based unweighted Unifrac distance matrices of OTU abundance profile.

DNA extracted from the vaginal specimens was subject to 16S rRNA gene amplicon sequencing and generated a total of 1,551,013 high-quality reads with an average of 7,716 ± 1,773 reads per sample. The reads were clustered into 367 operational taxonomic units (OTUs), resulting in a mean yield of 25.4 ± 18.9 OTUs per sample. On D0, all participants displayed clear dysbiosis in vaginal microbial composition, evidenced by low levels of *Lactobacillus* and apparent presence of many BV-associated microbes (e.g., *Aerococcus, Atopobium, Megasphaera*, and *Prevotella*, Figure 1B and Supplementary Figure 3). Immediately after the therapy (D7), the vaginal microbiota exhibited apparent alterations in the composition (Figures 1A,B) and diversity (Figure 1C) in comparison with those on D0. In addition, although both groups had similar microbial compositions on D0 and D7, they greatly differed on D30 (Figure 1B and Supplementary Figure 2). These findings demonostrated that the medication greatly affected the vaginal microbial composition.

Next, we examined the time-dependent dynamics of individual phylotypes in the two groups. At each time points, Firmicutes, Actinobacteria, Bacteroidetes, Fusobacteria, and Proteobacteria collectively accounted for over 95% of the relative abundance in both groups (Figure 1A). Each of the five taxa displayed clear time-dependent changes in relative abundance. Firmicutes, which was overall the most abundant phylum, displayed an apparent increase of relative abundance on D7 (>80%) in comparison with its levels on D0 (30–50%) in both recurrence and cure groups. On D30, however, its relative abundance manifested a divergence between the two groups: in the recurrence group, its proportion was greatly reduced to a level similar to that of D0, whereas in the cure group, its proportion remained high. Both Actinobacteria and Bacteroidetes displayed the opposite pattern: in comparison with their D0 levels, the relative abundances of the two taxa on D7 clearly decreased in both groups; but on D30, their proportions were similar to those on D0 in the recurrence group but remained low in the cure group. Another phylum that was clearly different between the two groups was Fusobacteria, which in the recurrence group had a considerable proportion on D0 and remained roughly stable on D7 and D30; however, in the cure group the phylum was barely detectable at the latter two time points. Unlike the aforementioned four phyla whose relative abundances on D0 were comparable between the two groups, Proteobacteria appeared to display a higher D0 relative abundance in the recurrence group (6%) than that in the cure group (1%); at latter two time points, its relative abundance was decreased in the recurrence group and increased in the cure group.

At genus level, the most abundant taxa included *Lactobacillus* and *Enterococcus* of Firmicutes, *Atopobium* of Actinobacteria, *Prevotella* of Bacteroides, and *Sneathia* of Fusobacteria. Their dynamics of relative abundances (Figure 1B and Supplementary Figure 1) coincided with that of their parent phyla (Figure 1A). Overall, our observation of the vaginal microbial composition revealed that on D30, the medication-resulted microbiota changes on D7 were largely maintained in the cure group but dissipated in the recurrence group. Similar patterns were also found for diversity (Figure 1C) and functional analyses (Figure 1D). Because metronidazole is effective against anaerobes but has little, if any, effects on lactobacilli, it is possible that the observed increase in the relative abundance of *Lactobacillus* merely reflects reduced levels of anaerobes while lactobacilli remained stable. Regardless, our results showed that good prognosis was correlated with a persistent increase in dominance of *Lactobacillus* as well as a persistent decrease in relative abundances of BV-associated microbes and microbial diversity.

### Examination of Clinical and Demographical Factors Associated With BV Recurrence

To assess the impact of clinical factors (Supplementary Table 1) on treatment outcome, League table chi-square test, rank sum test, or Fisher exact test was used to compare a set of parameters between the recurrent group and cure group. The results revealed that intrauterine device (IUD) use, past history of BV, and D7 Nugent score were associated with post-treatment recurrence (Table 1). On the other hand, smoking, menstrual status, vaginal pH on D0, severity of symptoms (i.e., “fishy” odor, discharge, and pruritus) on D0, history of uterine cavity surgery, and perioperative sexual behaviors (between D0 and D7 and between D7 and D30) showed no effects on BV recurrence. Our results were in agreement with some previous studies that disease history and Nugent score were associated with the treatment outcome of BV (Cherpes et al., 2008; Hay, 2009) and that different intrauterine devices were found to affect BV vulnerability (Madden et al., 2012).

**Table 1:** Association analysis of clinical factors and BV recurrence.

**Factor**	**Analysis method**	***P*-value**
Smoking	Fisher exact test	1
History of BV	Chisq.test	0.04053
History of surgery	Fisher exact test	0.1528
Use of intrauterine device	Chisq.test	0.03946
Menstrual status	Fisher exact test	0.1503
Nugent score	Fisher exact test	0.0004888
Sexual behaviors during D0-D7	Fisher exact test	0.721
Sexual behaviors during D7-D30	Fisher exact test	1
Vaginal “fishy” odor	Fisher exact test	0.756
Vaginal pruritus	Chisq.test	0.6423
Age	Rank sum test	0.4142
pH	Rank sum test	0.9123
Vaginal discharge	Chisq.test	0.1255

### Examination of Microbes Associated With BV Recurrence

**Figure 2:**
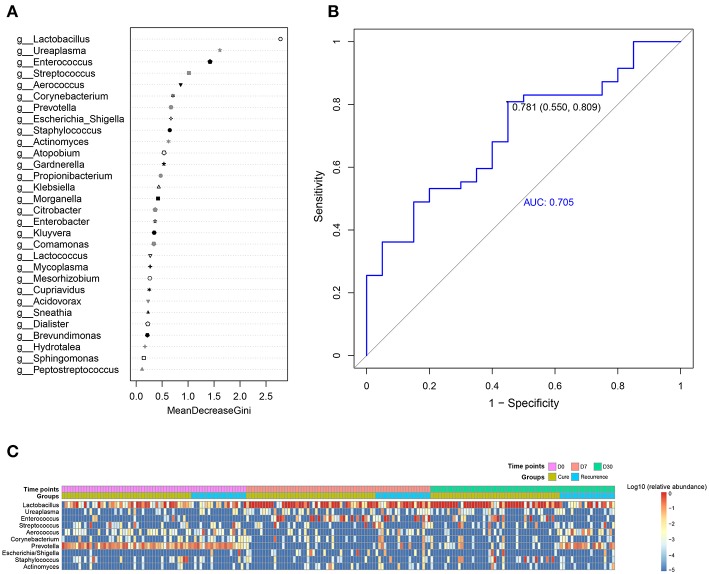
A predictive model of importance based on the genus-level abundance profile using random forests. **(A)** The relative importance of each genus in the predictive model was assessed using the mean decreasing Gini coefficient in vaginal microbiota. **(B)** The ROC curve for predicting recurrence generated by random forest; the plots shown in ROC represent the corresponding optimal threshold. **(C)** The heatmap of top 10 phylotypes for recurrence importance generated by random forest.

Next, we analyzed the association of individual vaginal genera with the treatment outcome. To test this, we used two different classification algorithms for importance ranking, namely random forest and Extreme Gradient Boosting (XGBoost) (Chen and Guestrin, 2016). Our rationale was that phylotypes of critical importance were likely identified by both methods, despite their divergent modeling approaches: random forest uses fully grown decision trees featuring low bias and high variance, whereas XGBoost employs weak learners characterized by high bias and low variance. The top 10 recurrence-associated genera identified by random forest, in descending order of importance, were *Lactobacillus, Ureaplasma, Enterococcus, Streptococcus, Aerococcus, Corynebacterium, Prevotella, Escherichia*_*Shigella, Staphylococcus*, and *Actinomyces* (Figure 2A), whereas those identified by XGBoost were *Sneathia, Lactobacillus, Ureaplasma, Enterococcus, Aerococcus, Escherchia*_*Shigella, Gardnerella, Staphylococcus, Prevotella*, and *Corynebacterium* (Supplementary Figure 3A). The two groups of microbes were similar in composition and ranking; 8 genera were identified by both methods, which were *Lactobacillus, Enterococcus, Ureaplasma, Aerococcus, Corynebacterium, Prevotella, Escherichia*_*Shigella*, and *Staphylococcus*. The performance of random forest classification or XGBoost based on the most discriminatory OTUs resulted in an area under the ROC curve (AUC) of 0.706 or 0.985 for predicting recurrence using D7 data (Figure 2B and Supplementary Figure 3B). Interestingly, whereas many of these phylotypes important for recurrence, such as *Lactobacillus, Enterococcus, Aerococcus, Prevotella, Escherichia*_*Shigella, Staphylococcus*, and *Sneathia*, exhibited noticeable differences of relative abundance between the cure and recurrence groups on D30 and/or D7, others (e.g., *Actinomyces, Ureaplasma, Corynebacterium*) did not show such inter-group distinction (Figures 1B, 2C and Supplementary Figure 2). Moreover, many phylotypes exhibited apparent inter-group differences on D30, including *Atopobium, Dialister, Gemella, Magasphaera, Parvimonas*, and *Peptoniphilus* (Supplementary Figure 2), were not identified to be top recurrence-associated taxa. These findings indicated that the recurrence association was not a simple reflection of relative abundance dynamics. In summary, our findings showed that the phylotypes most important for the treatment outcome could be the beneficial keystone bacteria of *Lactobacillus*, or BV-associated microbes such as *Aerococcus, Prevotella*, and *Staphylococcus*.

### Co-occurrence Network Revealed Extensive Interbacterial Associations

**Figure 3:**
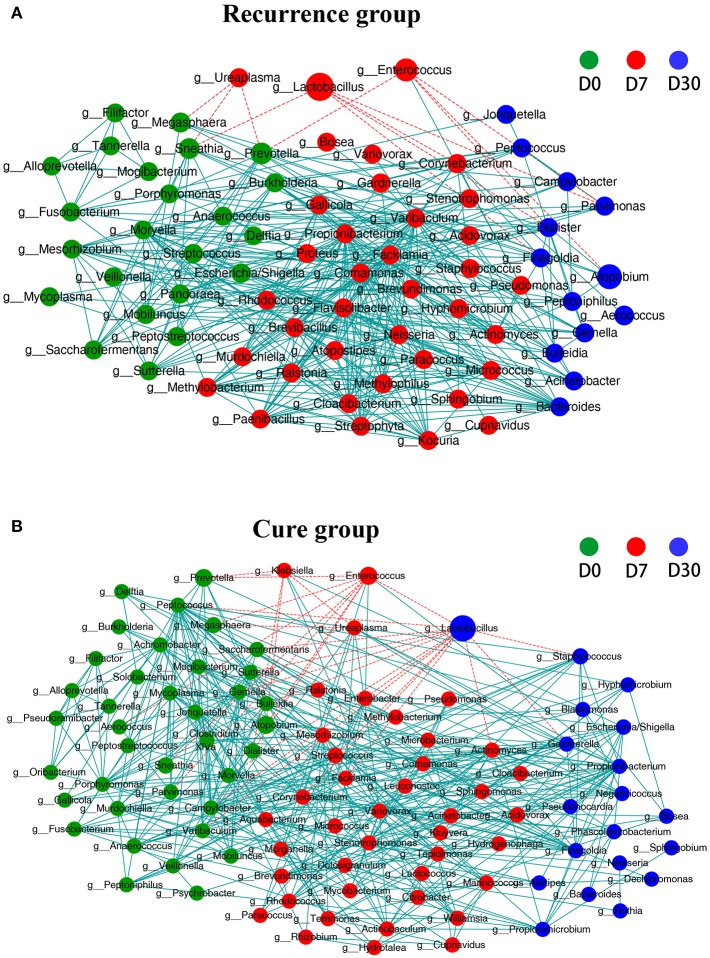
Co-occurrence network showing the correlations between major phylotypes of vaginal microbiota at different time points in the recurrence group **(A)** and cure group **(B)**. Each genus is only shown in a color corresponding to the time point when it has the highest relative abundance. Node size indicates the average abundance of each genus. Lines between nodes represent the interbacterial correlations (edges), and blue solid line and red dashed line indicate positive and negative correlations, respectively.

Co-occurrence networks were constructed and used to examine interbacterial correlations in the two clinical groups. The results (Figure 3) illustrated the extremely complex network of interbacterial interactions that accommodated BV-associated genera including *Sneathia, Megasphaera, Prevotella, Atopobium, Aerococcus*, and *Gardnerella*. The overwhelming majority of these interbacterial correlations (edges) are positive (blue, solid lines), indicating that there were considerable synergistic effects between various vaginal bacteria and that such positive association likely contributes to their survival and persistence in the hostile environment. Negative correlations (red, dashed lines) were mostly connected to *Lactobacillus* and *Enterococcus*. *Lactobacillus* is well-known for lactic acid production and suppression of other vaginal bacteria (Ma et al., 2012; Miller et al., 2016), which are consistent with these negative interbacterial interactions. *Enterococcus* is another genus of lactic acid bacteria. Negative interactions were also found for *Ureaplasma* (mostly in the recurrence group) and *Klebsiella* (mostly in the cure group). Interestingly, *Lactobacillus, Enterococcus*, and *Ureaplasma* all manifested increases in relative abundance on D7, as opposed to many other genera (Figures 1B, 2C and Supplementary Figure 2), indicating negative associations between the three genera and many BV-associated microbes. A comparison between the recurrence group and cure group also showed that the number of negative correlation edges, mostly from *Lactobacillus* and *Enterococcus*, were apparently less in the recurrence group than the cure group (12 vs. 24 negative edges), suggesting a probable connection between good prognosis and increased interbacterial suppression, particularly from *Lactobacillus* and *Enterococcus*.

### Changes of *Lactobacillus* Species Composition in the Two Groups and Generation of a 5-Component Predictive Model for Recurrence

**Figure 4:**
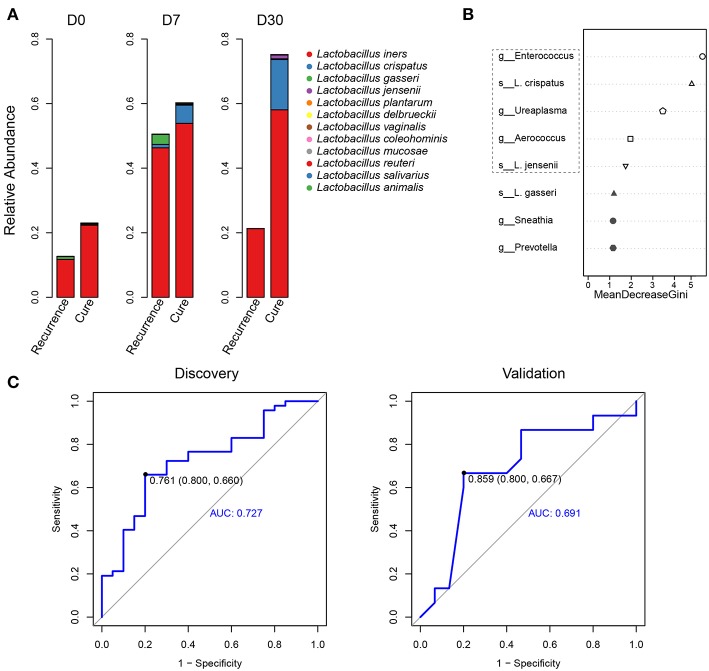
Species composition of *Lactobacillus* of vaginal microbiota at different time points in the recurrence group and cure group and generation of a 5-component panel for predicting recurrence. **(A)** The relative abundances of individual *Lactobacillus* species. **(B)** The relative importance of each phylotype in the predictive model was assessed using mean decreasing Gini coefficient in a pool of 8 candidates; the top 5 phylotypes in the box of the dashed line were included in the 5-component panel for recurrence prediction. **(C)** The ROC curve for predicting recurrence using the 5-component panel; the plots shown in ROC represent the corresponding optimal threshold.

Given the keystone roles of lactobacilli in maintaining vaginal health (Ma et al., 2012; Miller et al., 2016) and considerable biological differences between the different species (Ravel et al., 2011), we were curious about whether there were variations in recurrence-correlation of individual *Lactobacillus* species. To this end, we employed a previously reported approach (Ravel et al., 2011) to analyze the relative abundance changes of individual *Lactobacillus* species over the course of treatment. The results revealed that although *L. iners* remained the predominant *Lactobacillus* species, the cure group and recurrence group displayed apparent differences in *Lactobacillus* species composition (Figure 4A). Specifically, *L. crispatus, L. gasseri*, and *L. jensenii* manifested clearly different dynamics in the course of the treatment. On D0, the lactobacilli in the cure group were almost exclusively *L. iners*, whereas that in the recurrence group also had a detectable proportion of *L. gasseri*. On D7, *L. crispatus* was the only other *Lactobacillus* species in the cure group with a clear presence besides *L. iners*; whereas in the recurrence group, *L. gasseri* was more pronounced, although *L. crispatus* was also detectable. On D30, the cure group maintained a significant level of *L. crispatus*; whereas the recurrence group barely contained any non-*L. iners Lactobacillus* species. As such, our results revealed that relative abundances of several non-*L. iners Lactobacillus* species, mainly *L. crispatus, L. gasseri*, and *L. jensenii*, differred between the recurrence and cure groups.

Given that our results had identified several discriminatory phylotypes for BV recurrence, we attempted to develop a small panel of microbes for predicting recurrence using data on D7 (when a typical metronidazole therapy ends). We first established a pool of candidates comprising the following phylotypes: the three *Lactobacillus* species of *L. crispatus, L. gasseri*, and *L. jensenii*; *Enterococcus, Ureaplasma*, and *Aerococcus*, all being top 5 recurrence-associated genera based on both random forest and XGBoost (Figure 2 and Supplementary Figure 3); *Prevotella*, a top 10 recurrence-associated genus based on both random forest and XGBoost that displayed apparent inter-group changes of relative abundance (Figures 1, 2 and Supplementary Figure 3); *Sneathia*, the top recurrence associated genus according to XGBoost that displayed apparent inter-group changes of relative abundance (Figures 1, 2). Random forest analysis on the 8 phylotypes revealed that *Enterococcus, L. crispatus, Ureaplasma, Aerococcus*, and *L. jensenii* were the top 5 components for recurrence association (Figure 4B). Application of this 5-component panel using D7 data to predict recurrence yielded the specificity, sensitivity, and AUC values of 0.80, 0.66, and 0.73 for the discovery cohort and 0.80, 0.67, and 0.69 for the validation cohort (Figure 4C), respectively.

## Discussion

BV-associated microbes vary considerably from person to person and may include species in *Gardnerella, Atopobium, Prevotella, Sneathia, Peptostreptococcus, Mobiluncus, Leptotrichia, Mycoplasma* and BV-associated bacterium 1 (BVAB1) to BVAB3 (Fredricks et al., 2005; Onderdonk et al., 2016). The fact that BV is a dysbiotic condition associated with many microbes possibly contribute to its significant recurrence rate, as the treatments do not target a specific bacterium or a few conserved ones (Sobel et al., 2006). Currently, the reported recurrence rates of metronidazole or clindamycin treatment of BV were between 10–50% at 1-to-3 months (Fischbach et al., 1993; Livengood et al., 1999; Koumans et al., 2007; Bunge et al., 2009; Xiao et al., 2016), which are in line with the recurrence rate (30% at 1 month) found in this study.

Microbial persistence is affected by multiple host factors. A 1-year study examining 121 Australian BV patients showed that the recurrence was associated with a past history of BV, a regular sex partner, and female sex partners (Bradshaw et al., 2006). In addition, a large cohort study examining BV susceptibility showed that increased incidence of BV was associated with ethnicity, cigarette smoking, vaginal intercourse, Nugent score, use of intrauterine device, and unconventional sexual behaviors (Cherpes et al., 2008). In this study, our results revealed that past BV history, use of intravaginal device, and D7 Nugent score were significantly associated with BV recurrence in a Chinese cohort; the findings were overall in line with these previous reports (Bradshaw et al., 2006; Cherpes et al., 2008; Madden et al., 2012).

Clinical studies based on adjuvant application of probiotics showed that some *Lactobacillus* species/isolates augmented the cure rates of antibiotics-based therapy (Neri et al., 1993; Shalev et al., 1996; Anukam et al., 2006), but other reports found no such effects (Fredricsson et al., 1989; Eriksson et al., 2005). The discrepancy could in part be due to the different species/strains used. In addition, it should be noted that these studies did not examine the indigenous lactobacilli or other microbes of vaginal microbiota, many of which likely have considerable influence on prognosis. In this study, we focused on the native vaginal microbiota dynamics in BV patients undergoing standard therapy and showed that *Lactobacillus* was the genus significantly associated with treatment outcome (Figure 2 and Supplementary Figure 3) and had negative interactions with many other microbes (Figure 3). This corroborated the fact that *Lactobacillus* is the keystone group in maintaining vaginal health (Ma et al., 2012; Miller et al., 2016). Lactic acid produced by vaginal microbiota is pivotal in suppressing pathogenic microbes and lactobacilli are the major producers of this compound on normal vaginal epithelium (Ma et al., 2012; Miller et al., 2016). It was reported that different *Lactobacillus* species exhibit considerable differences in lactic acid production and that *L. crispatus* is the most prolific contributor (Ma et al., 2012; Miller et al., 2016). In addition, *L. crispatus*-dominated vaginal microbiota or CST I community-state type I (CST I) (Ravel et al., 2011) is strongly associated with absence of BV (DiGiulio et al., 2015) or potent suppression of HIV (Nunn et al., 2015); similar pattern of *L. crispatus* dominance in healthy people was also found in urinary microbiota (Gottschick et al., 2017a). A large-cohort analysis on vaginal microbiota (Ravel et al., 2011) revealed that although *Lactobacillus* species all displayed negative associations with Nugent score, the degrees of negative association differred: that of *L. crispatus* and *L. jensenii* were comparable with each other but higher that of *L. iners*, which was followed by *L. gassei*. Interestingly, this ranking of Nugent score association coincides with the differential relative abundances of the four *Lactobacillus* species in the two treatment groups (Figure 4A), where only *L. crispatus* and *L. jensenii* were enriched in the cure group on D7 and/or D30. These findings collectively indicated that the species dynamics of *Lactobacillus*, rather than the genus as a whole, is more informative for monitoring the treatment outcome.

*Lactobacillus* belongs to the order of Lactobacillales (lactic acid bacteria, LAB), which also includes *Enterococcus, Aerococcus*, among others and many are linked to food fermentations. Interestingly, our results indicated that *Enterococcus* behaved similarly as *Lactobacillus* in relative abundance (Figure 1B), recurrence association (Figure 2A), and interbacterial interactions (Figure 3). However, at sample level, *Enterococcus* exhibited a quite different dynamics from that of *Lactobacillus* (Figure 2C and Supplementary Figure 4), which was particularly apparent in both groups on D7, when close to 40 samples contained a majority (>50%) of *Lactobacillus* but minimal (<5%) *Enterococcus*, whereas 15 samples had a clear presence (>30%) of *Enterococcus* but minimal *Lactobacillus* (<5%); this lopsided pattern persisted in the cure group on D30. We speculated that during the treatment, *Enterococcus* can suppress other bacteria, which perhaps involves lactic acid secretion, and is therefore crucial for patients lacking an adequate level of lactobacilli.

*Ureaplasma* was another major recurrence-associated phylotype with multiple negative interbacterial interactions. Unlike *Lactobacillus* and *Enterococcus, Ureaplasma* showed negative interabacterial interactions only in the recurrence group (Figure 3), suggesting that the genus suppresses other microbes more efficiently in this group of patients. Although not commonly associated with BV, *Ureaplasma* species are highly prevalent colonizers in urogenital areas and can cause inflammations (Marovt et al., 2015; Vancutsem et al., 2015; Sweeney et al., 2017). Unlike most bacteria, *Ureaplasma* species have very small genomes (0.75–0.95 Mbp) (Paralanov et al., 2012) that may contribute to their limited biosynthesis capacity, lack of cell wall, and obligate parasitic lifestyle (Combaz-Sohnchen and Kuhn, 2017). Hence, we argue that the ability of *Ureaplasma* to suppress other bacteria and affect recurrence (Figures 2, 3) is unlikely due to release of bactericidal compounds, as do *Lactobacillus* and *Enterococcus*. Rather, it is more plausible that the presence of *Ureaplasma* in the vagina can somehow provoke a robust host immune response, which leads to suppression of other bacteria. Multiple-banded antigen (MBA) of *Ureaplasma* is a major surface-exposed, immunodominant antigen that activates the NF-κB pathway and subsequent production of cytokines by signaling through Toll-like receptors 1, 2, and 6 (Shimizu et al., 2008). It will be interesting to see whether this protein or any other component of *Ureaplasma* is implicated in affecting recurrence and whether application of this protein can affect the persistence of BV-associated microbes.

Unlike *Lactobacillus* and *Enterococcus* that on D30 were enriched in the cure group, *Aerococcus* was depleted in this group; this pattern was similar to several other BV-associated bacteria such as *Prevotella, Atopobium, Dialister, Sneathia, Mobiluncus, Mycoplasma*, and *Megasphaera* (Supplementary Figure 3). It is interesting to notice that among the top 5 genera of recurrence importance identified by both random forest and XGBoost, *Aerococcus* was the only genus of BV-associated bacteria and ranked the lowest. In other words, the key phylotypes dictating the treatment outcome are more likely to be those suppressive of other bacteria than so-called BV-associated bacteria.

One caveat of our study was the low representation of *Gardnerella* in the discovery cohort but not in the validation cohort (Figure 1B and Supplementary Table 3). Of note, amplification of the V1-V3 region was used in the discovery cohort whereas that of the V3-V4 region was adopted for the validation cohort. As such, this result was reminiscent of the low proportions of this genus in BV patients reported in a previous study (Yeoman et al., 2013), which also employed V1-V3 amplification. Because amplification of the V1-V2 region, which shares the forward V1 primer, generated expected relative abundance data of *Gardnerella* (Gottschick et al., 2017b), the problem of poor *Gardnerella* detectability appeared to be caused by the reverse V3 primer.

In conclusion, this study generated the following findings. First, the dynamics of vaginal microbiota, specifically that of *Lactobacillus* and BV-associated microbes, coincided with the treatment outcome. Second, IUD use, past history of BV, and D7 Nugent score were significantly associated with recurrence. Third, *Lactobacillus, Enterococcus, Ureaplasma*, and *Aerococcus* were shown by both random forest analysis and XGBoost to be among the top 5 genera for recurrence importance. Fourth, *L. crispatus, L. gasseri*, and *L. jensenii* displayed very different dynamics of relative abundance over the course of treatment. Fifth, we developed a 5-component model composed of *Enterococcus, L. crispatus, Ureaplasma, Aerococcus*, and *L. jensenii* for predicting recurrence and generated a moderately good performance (AUC of 0.73 in the discovery cohort and 0.69 in the validation cohort). Although this study is limited by the small sample size, our findings argues that a small panel of key phylotypes may be developed to monitor the treatment outcome and in turn improve the prognosis.

## Ethics Statement

This study was carried out in accordance with the recommendations of Regulations of Clinical Trials issued by China Food and Drug Administration. Written informed consent in accordance with the Declaration of Helsinki was obtained from all subjects. This study was approved by the Ethics Committee of Peking University First Hospital, Beijing, China.

## Author Contributions

BX, QX, and CW designed the project. QX and BX managed the project. BX, XN, and ZL collected samples and performed the clinical study. XN performed DNA extraction experiments. QX performed library construction and sequencing. BX, QX, WS, and CW designed the analysis and analyzed the data. BX, QX, and CW wrote the paper. QX, CW, and NQ revised the manuscript.

### Conflict of Interest Statement

QX is employed by Shenzhen Jinrui Biotechnology, Co., Ltd. The remaining authors declare that the research was conducted in the absence of any commercial or financial relationships that could be construed as a potential conflict of interest.

## Supplementary Material

Supplementary Figure 1 Flowchart illustrating the recruitment, treatment, and data collection of the patients with BV.

Supplementary Figure 2 The heatmap of all genera identified in this study.

Supplementary Figure 3 A predictive model of importance based on the genus-level abundance profile using XGBoost. **(A)** The relative importance of each genus in the predictive model. **(B)** The ROC curve for predicting recurrence.

Supplementary Figure 4 The dynamics of relative abundance of *Lactobacillus* and *Enterococcus* in individual participants.

Supplementary Table 1 Demographic and clinical traits of the cure and recurrence groups of the discovery cohort.

Supplementary Table 2 The clinical records of the validation cohort.

Supplementary Table 3 The sequencing results of Gardneralla of the discovery and validation cohorts.
