# Supplementary figures and images for: Association Analysis on Recurrence of Bacterial Vaginosis Revealed Microbes and Clinical Variables Important for Treatment Outcome
Source: Front Cell Infect Microbiol. 2019 Jun 11;9:189. doi: 10.3389/fcimb.2019.00189 (PMC6579829; doi:10.3389/fcimb.2019.00189)

# Study design

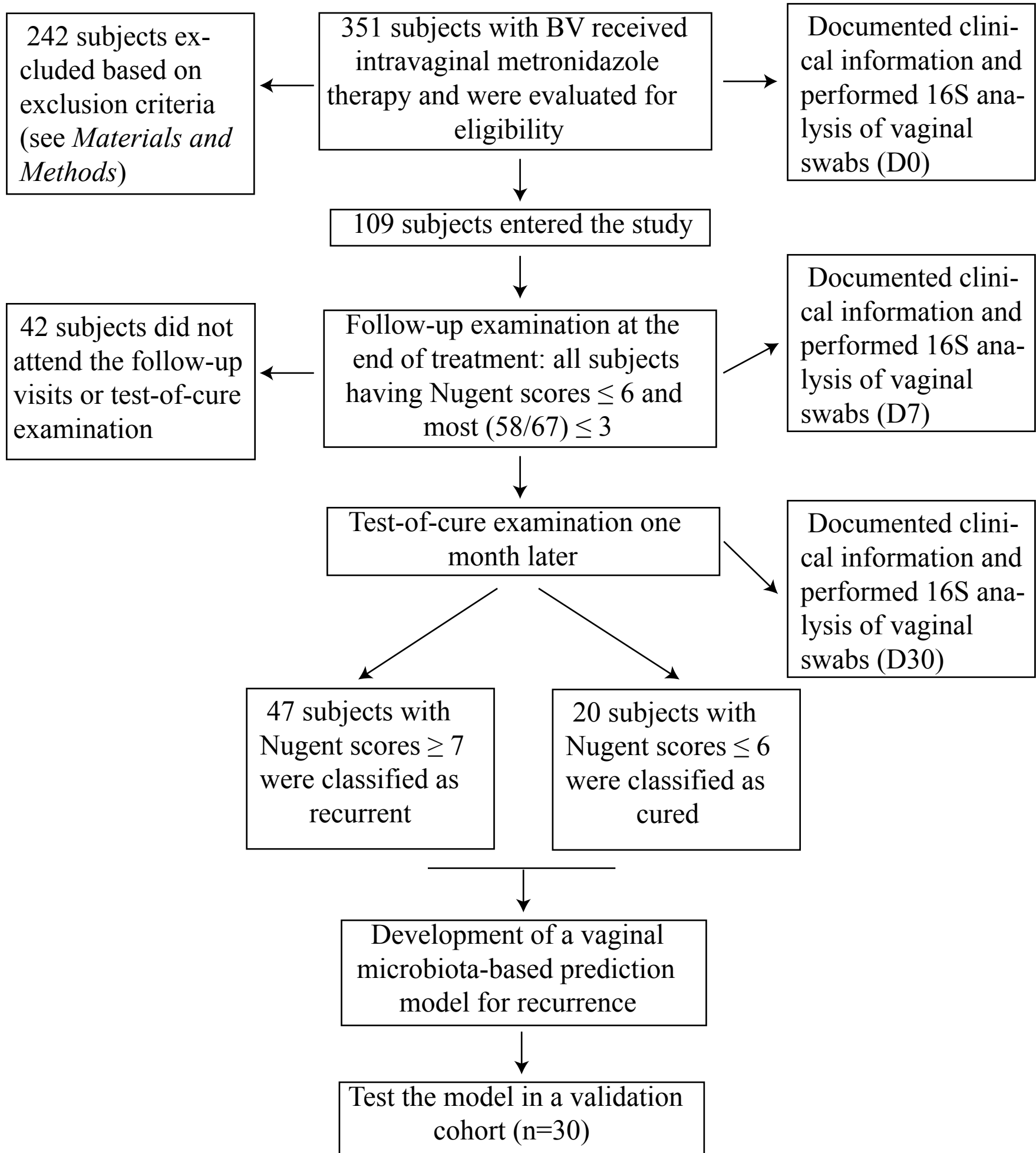

Supplement: Supplementary Figure 1 — Flowchart illustrating the recruitment, treatment, and data collection of the patients with BV. [file Data_Sheet_1.PDF]

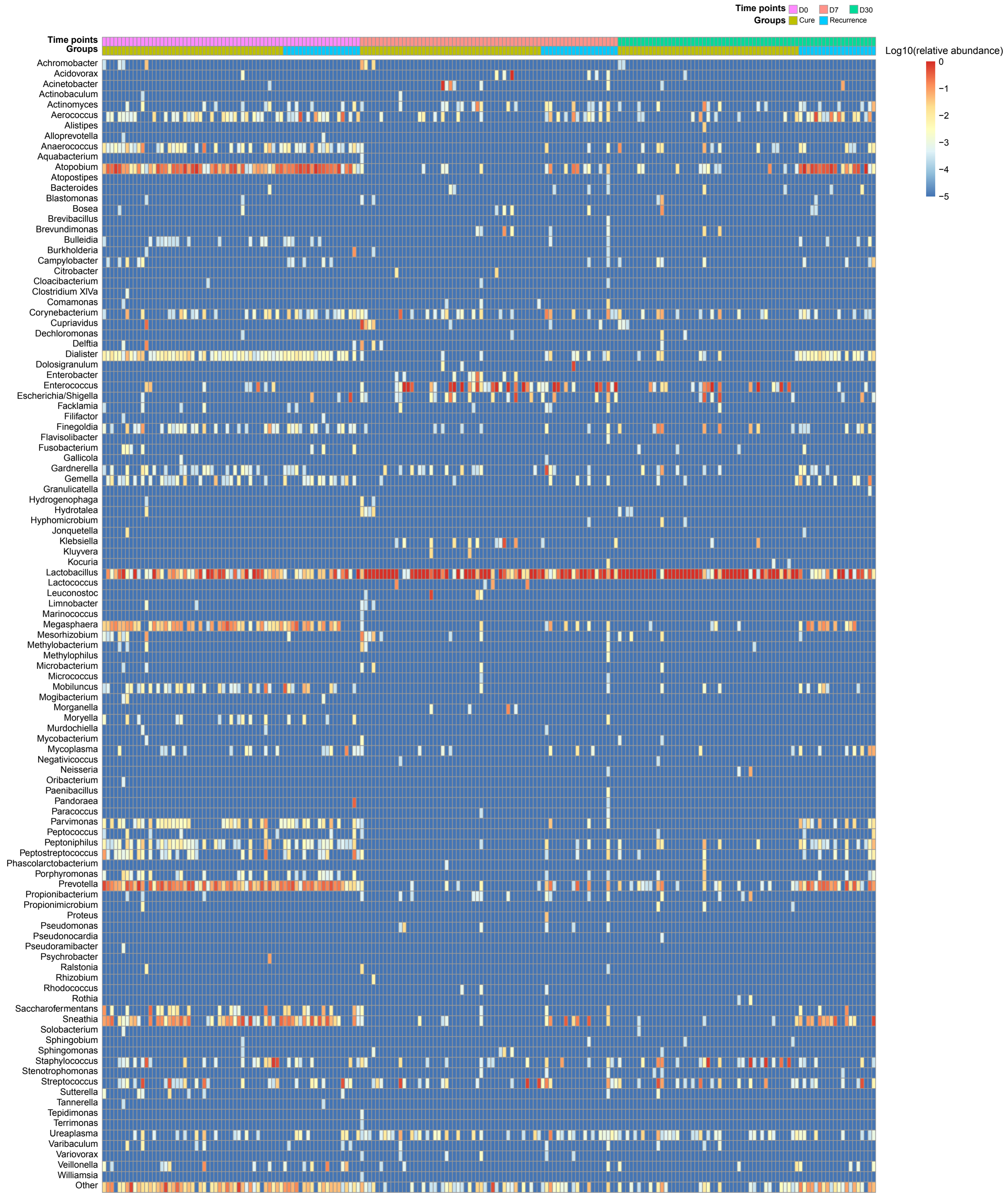

Supplement: Supplementary Figure 2 — The heatmap of all genera identified in this study. [file Data_Sheet_2.PDF]

A

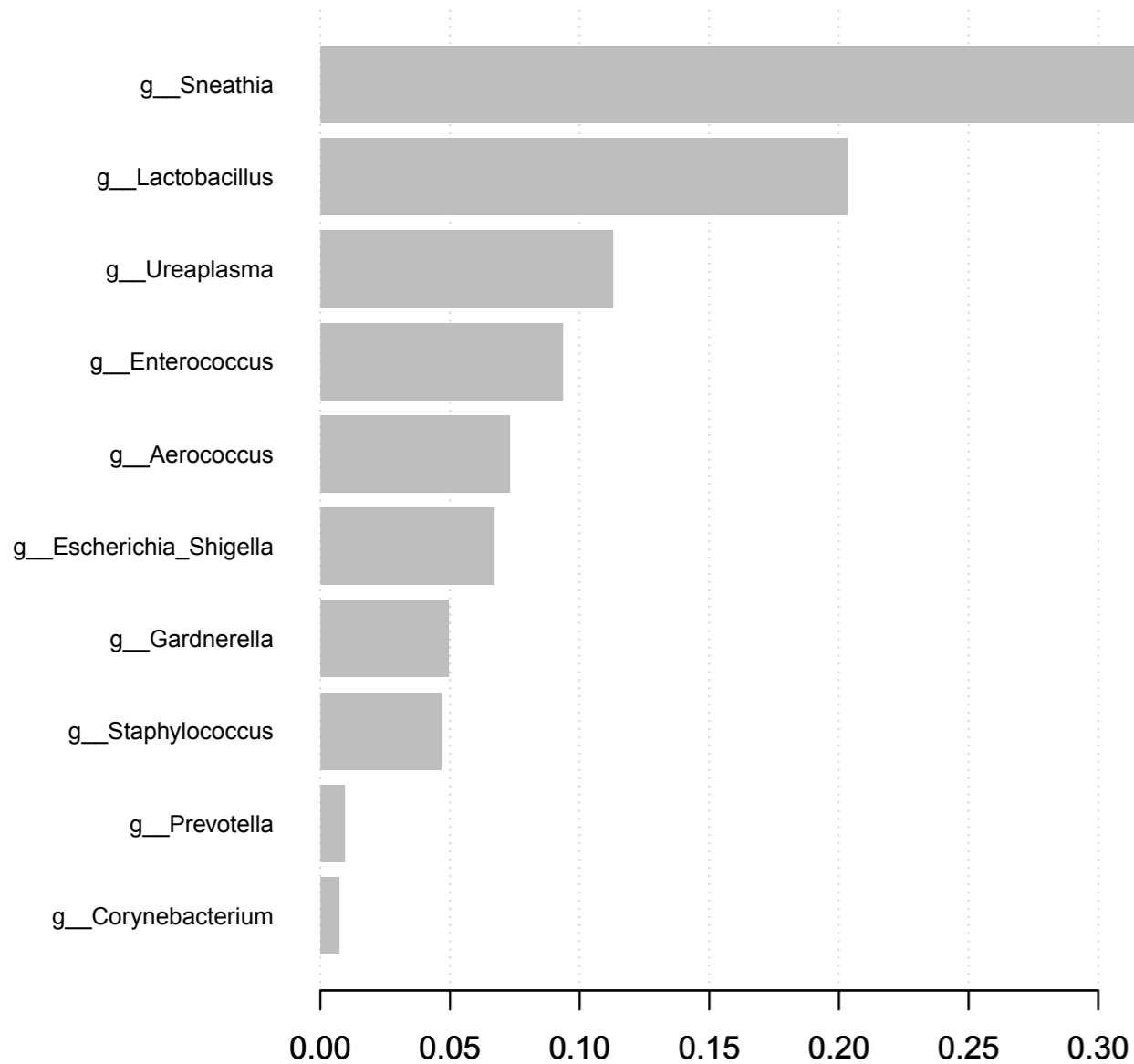

B

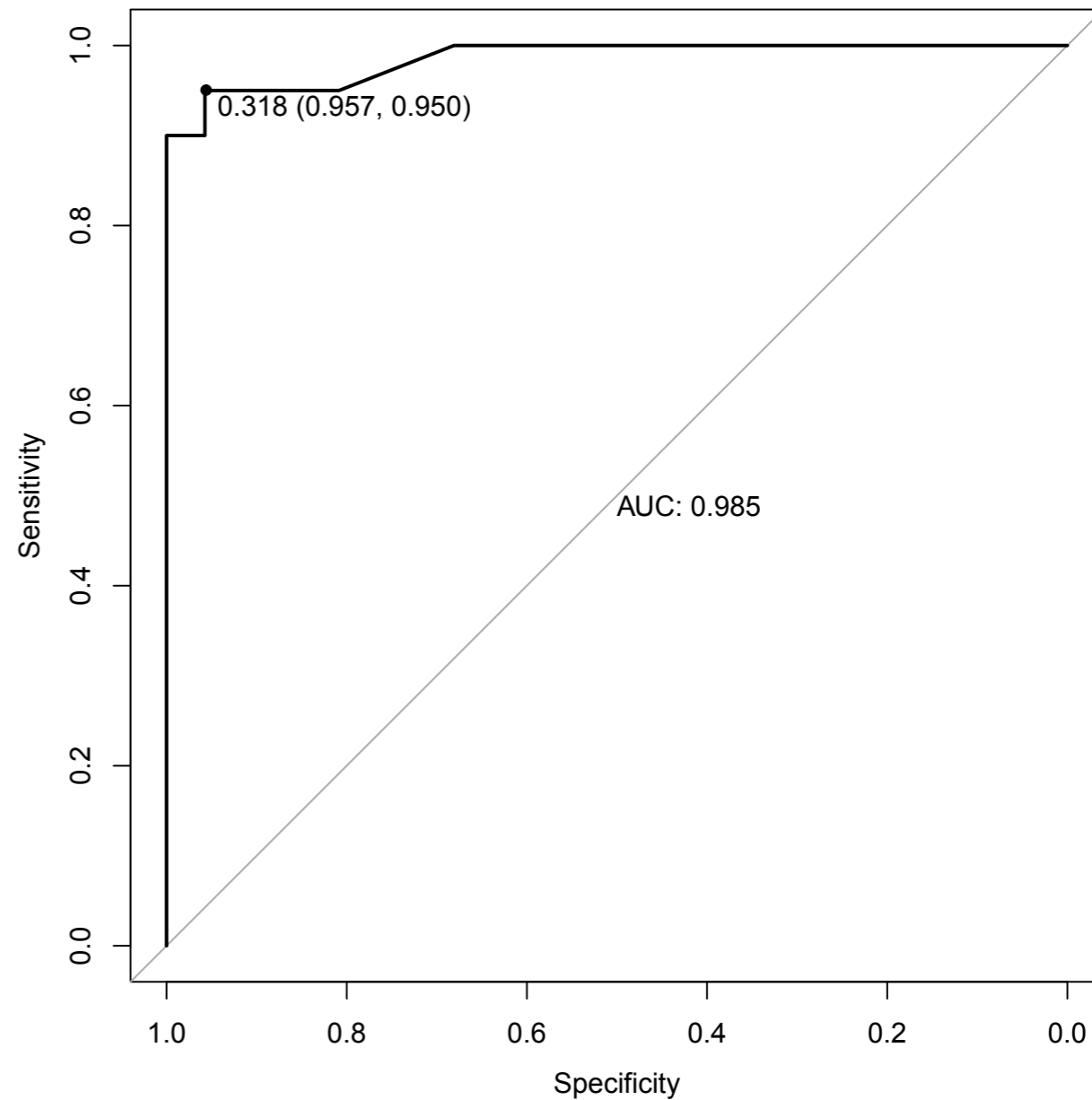

Supplement: Supplementary Figure 3 — A predictive model of importance based on the genus-level abundance profile using XGBoost. (A) The relative importance of each genus in the predictive model. (B) The ROC curve for predicting recurrence. [file Data_Sheet_3.PDF]

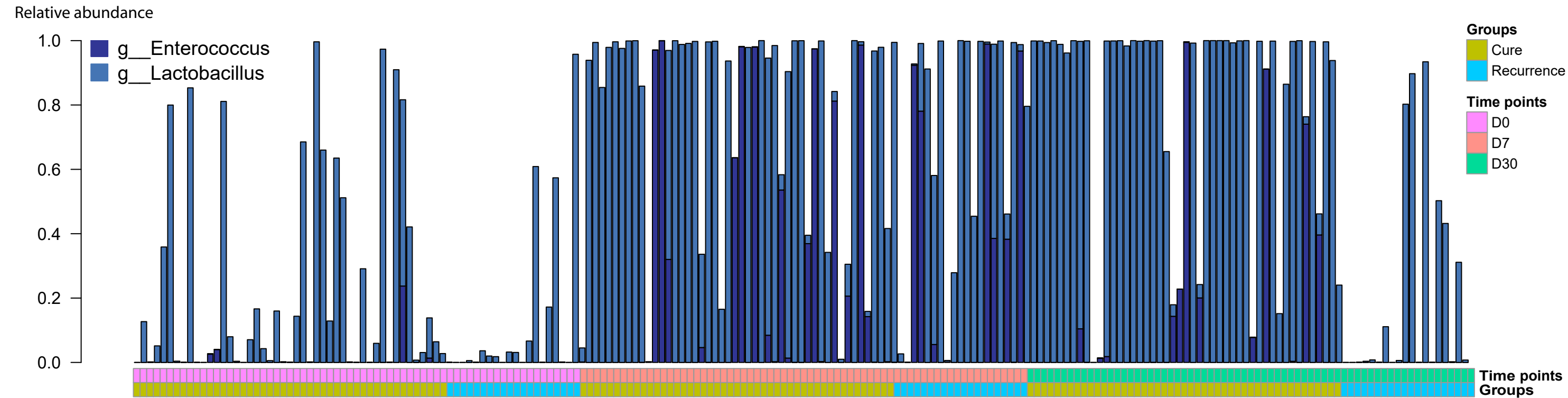

Supplement: Supplementary Figure 4 — The dynamics of relative abundance of Lactobacillus and Enterococcus in individual participants. [file Data_Sheet_4.PDF]
