# Supplementary material for: Association Analysis on Recurrence of Bacterial Vaginosis Revealed Microbes and Clinical Variables Important for Treatment Outcome
Source: Front Cell Infect Microbiol. 2019 Jun 11;9:189. doi: 10.3389/fcimb.2019.00189 (PMC6579829; doi:10.3389/fcimb.2019.00189)

Supplementary Table 1. Demographic and clinical traits of the cure and recurrence groups of the discovery cohort.


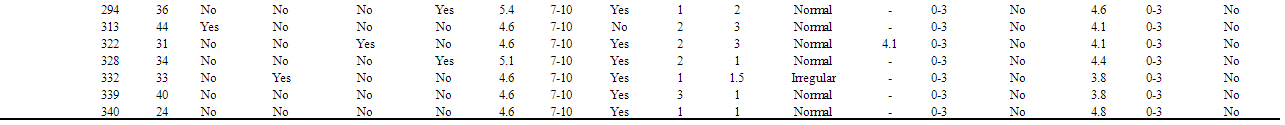

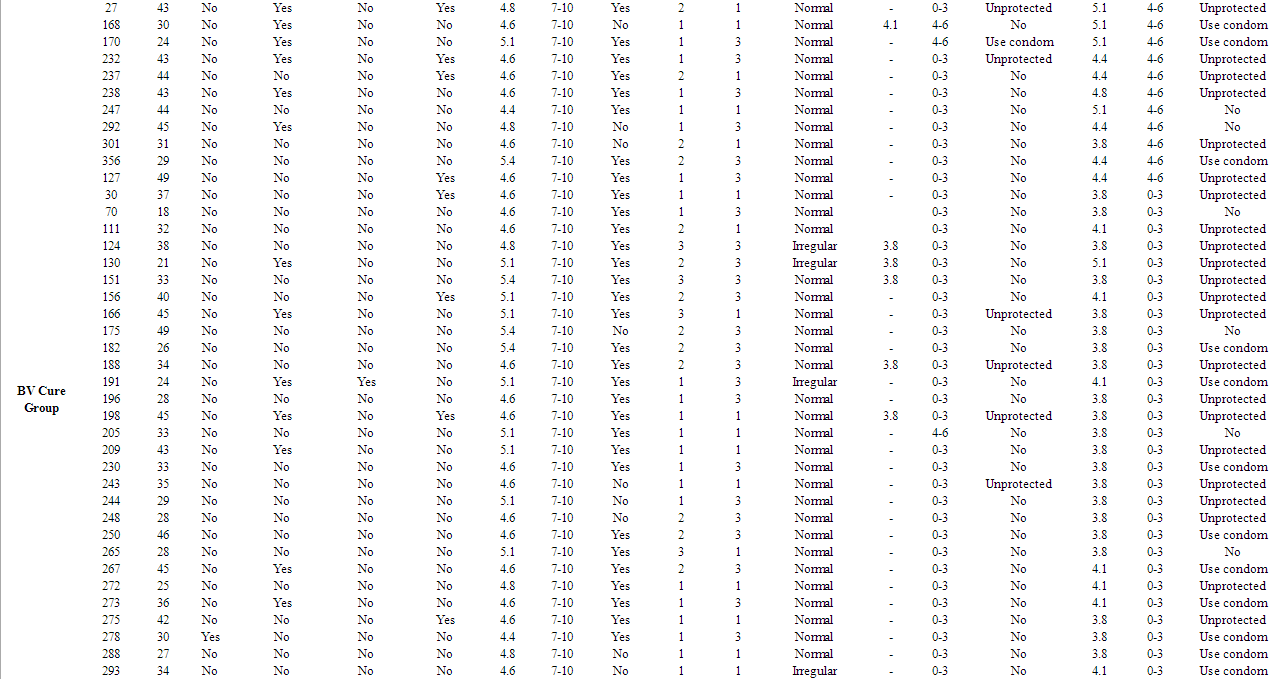

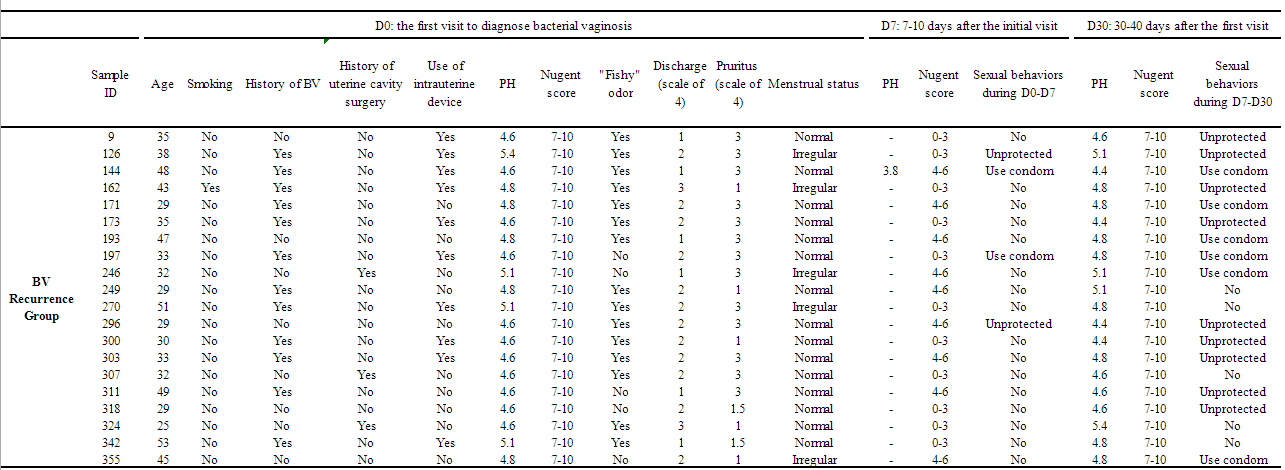

Supplement: Supplementary Table 1 — Demographic and clinical traits of the cure and recurrence groups of the discovery cohort. [file Table_1.DOCX]
